# Target Complexity Modulates Syntactic Priming During Comprehension

**DOI:** 10.3389/fpsyg.2020.00454

**Published:** 2020-03-18

**Authors:** Samar Husain, Himanshu Yadav

**Affiliations:** ^1^Department of Humanities and Social Sciences, Indian Institute of Technology Delhi, New Delhi, India; ^2^Department of Linguistics, University of Potsdam, Potsdam, Germany

**Keywords:** syntactic priming, top-down parsing, sentence comprehension, SOV language, Hindi

## Abstract

Syntactic priming is known to facilitate comprehension of the target sentence if the syntactic structure of the target sentence aligns with the structure of the prime (Branigan et al., 2005; Tooley and Traxler, 2010). Such a processing facilitation is understood to be constrained due to factors such as lexical overlap between the prime and the target, frequency of the prime structure, etc. Syntactic priming in SOV languages is also understood to be influenced by similar constraints (Arai, 2012). Sentence comprehension in SOV languages is known to be incremental and predictive. Such a top-down parsing process involves establishing various syntactic relations based on the linguistic cues of a sentence and the role of preverbal case-markers in achieving this is known to be critical. Given the evidence of syntactic priming during comprehension in these languages, this aspect of the comprehension process and its effect on syntactic priming becomes important. In this work, we show that syntactic priming during comprehension is affected by the probability of using the prime structure while parsing the target sentence. If the prime structure has a low probability given the sentential cues (e.g., nominal case-markers) in the target sentence, then the chances of persisting with the prime structure in the target reduces. Our work demonstrates the role of structural complexity of the target with regard to syntactic priming during comprehension and highlights that syntactic priming is modulated by an overarching preference of the parser to avoid rare structures.

## 1. Introduction

Syntactic priming (or syntactic persistence) has been one of the most enduring effects attested in the sentence processing literature. It was first experimentally demonstrated by a series of experiments by Bock (1986). Using a picture description task, Bock (1986) showed that English native speakers were systematically influenced by the abstract syntactic structure of an unrelated sentence they read out loud, prior to attending a picture. Since this landmark work, syntactic priming has been shown in numerous studies (Bock, 1989; Bock and Loebell, 1990; Bock et al., 1992; Pickering and Branigan, 1998; Ferreira, 2003; Scheepers, 2003). It has been shown to occur for a variety of constructions (Hartsuiker et al., 1999; Hartsuiker and Westenberg, 2000; Cleland and Pickering, 2003), and with a variety of experimental paradigms (Pickering and Branigan, 1998; Potter and Lombardi, 1998; Branigan et al., 2000; Hartsuiker and Westenberg, 2000); see, Pickering and Ferreira (2008) and Mahowald et al. (2016), for an overview. More importantly, in the context of the current work, syntactic priming has been attested cross-linguistically (e.g., Hartsuiker and Kolk, 1998; Scheepers, 2003), including languages that are typologically different from English, e.g., SOV languages such as Korean, Japanese, etc. (Shin and Christianson, 2009; Tanaka et al., 2009; Santesteban et al., 2015); see, Arai (2012) for an overview. For example, using a picture description task, Tanaka et al. (2009) showed that Japanese native speakers were more likely to describe a picture using a passive structure if they had been exposed to a passive prime such as 1b, compared to seeing an active prime such as 1a.


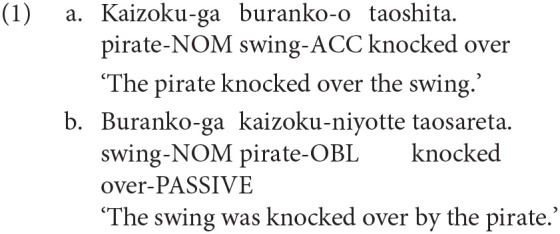


Syntactic priming has been demonstrated extensively during language production tasks (see, Mahowald et al., 2016), however recent work suggests that syntactic priming can also be observed during sentence comprehension (Pickering and Traxler, 2004; Branigan et al., 2005; Arai et al., 2007; Ledoux et al., 2007; Thothathiri and Snedeker, 2008a; Traxler, 2008; Traxler and Tooley, 2008; Tooley et al., 2009; Weber and Indefrey, 2009). For example, Arai et al. (2007) recorded participants' eye-movements on a picture displayed on a monitor while they heard sentences such as 3a, 3b. They found that participants were more likely to fixate on ‘the princess' when they heard the verb (‘send'), if 2a was read before. Similarly, after reading 2b, the participants were more likely to fixate on the picture of a necklace after hearing the verb (‘send') in 3b. This demonstrates that the comprehension process was being influenced by the exposure to the syntactic structure read in the immediate past.


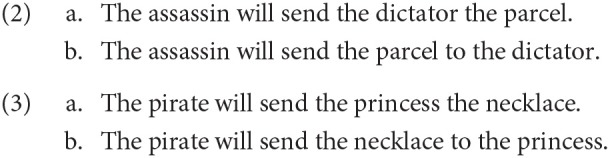


Syntactic priming during comprehension is now an established phenomenon (Tooley and Traxler, 2010). Again, priming during comprehension has been demonstrated in SOV languages as well (Tanaka et al., 2007; Arai and Mazuka, 2010). For example, in a study by Arai and Mazuka (2010), participants heard sentences such as 4a and 4b while looking at a picture in a visual world paradigm experiment. 4a is an active sentence while 4b is a passive sentence in Japanese. Prior to this, participants were exposed to a prime sentence which either matched the structure of the target or not. The results showed that participants made more anticipatory eye-movements to the agent when the prime was passive compared to when it was active. Note that *Sarusan-ga* ‘monkey-NOM' was role ambiguous in the target picture. The eye-movements to the agent before the disambiguating region therefore shows that participants were expecting to hear a passive construction after a passive prime.


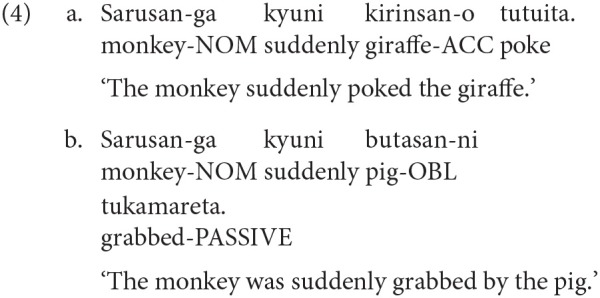


The above effects were observed before the critical verb in the target sentence was heard. This suggests that priming in SOV languages could be lexically independent. However, it has been suggested that the role of lexical overlap is critical for syntactic priming in comprehension. For example, in the Arai et al. (2007) study discussed earlier, the priming effect observed in the form of anticipatory eye-movements at the verb was found only when the verb form in the prime and the target sentences was identical. In general, many studies have highlighted the necessity of such a lexical overlap between prime and target as a precondition for syntactic priming during sentence comprehension (but see, Thothathiri and Snedeker, 2008a,b; Traxler, 2008).

To summarize, evidence for syntactic priming during comprehension in SOV languages or in SOV constructions (e.g., Hartsuiker and Westenberg, 2000) have been attested in numerous research. In all these studies we see a facilitation during comprehension due to this phenomenon. Critically, the facilitation due to priming is understood to happen when there is a lexical overlap between the prime and the target. Such a pattern can easily be explained by the Residual Activation Theory (Pickering and Branigan, 1998). Pickering and Branigan (1998) propose a network model to encode syntactic information of verbs–verb lemma nodes are connected to a category node, featural nodes as well as combinatorial nodes. Syntactic priming is explained as higher activation of the combinatorial node connected to the prime verb lemma node. In the case of priming due to lexical overlap, it is assumed that in addition to the combinatorial node, the link between the lemma and the combinatorial node is also activated. For example, Pickering and Branigan (1998) propose that reading a prime sentence like ‘The architect handed the latest plan over to the builder' activates the word form node, the lemma node and the combinatorial node associated with the verb ‘showed.' When a participant is then asked to complete a sentence fragment like ‘The patient showed …,' she is more likely to complete the sentence with the structure of the prime, such as ‘The patient showed the wound to the doctor' than completing it with an alternative structure like “The patient showed the doctor the wound.' It is assumed that processing the prime activates these network representations, and the residual activation of these nodes (and links) makes them accessible during a subsequent production task. Critically, the Pickering and Branigan (1998) residual activation theory can also explain priming during comprehension (see, Tooley and Traxler, 2010; Arai, 2012).

With regard to sentence comprehension in SOV languages, another factor that has been implicated during processing is the role of preverbal linguistic material in making robust prediction of the upcoming verbal head (e.g., Konieczny, 2000; Vasishth and Lewis, 2006; Nakatani and Gibson, 2010; Vasishth et al., 2010; Levy and Keller, 2013). In particular, it has been found that preverbal case-marked nouns in SOV languages provide constraining information to make the upcoming structure more precise, thus leading to facilitation during comprehension (e.g., Levy, 2008; Konieczny, 2000). For example, Husain et al. (2014) showed that, in Hindi, the relative clause verb (*parhi thii* ‘read PAST') in condition 5a was read faster than the verb in 5b. This slowdown was argued to be due to the robust use of the Ergative case-marker (=*ne*) on the relative pronoun (*jisne*) during the comprehension process. In particular, the prediction of the object preceding a verb was not met in condition 5b, while this prediction was met in condition 5a. Put differently, the slowdown at the relative clause verb reflected the cost of dashed expectation due to not encountering the expected canonical order (Subject-Object-Verb) in 5b where the relative clause had a non-canonical SVO order. The results showed that native speakers of Hindi were making predictions regarding the upcoming syntactic structure and when those predictions were not met, processing suffered. This pattern can be accounted by the information theoretic metric, surprisal (Hale, 2001; Levy, 2008). Surprisal equates the processing difficulty during comprehension with the conditional probability of encountering a word given the previous sentential context. The higher this probability (leading to a low surprisal value), the more likely the word will be to appear in the utterance, thereby making the processing at the word easy.


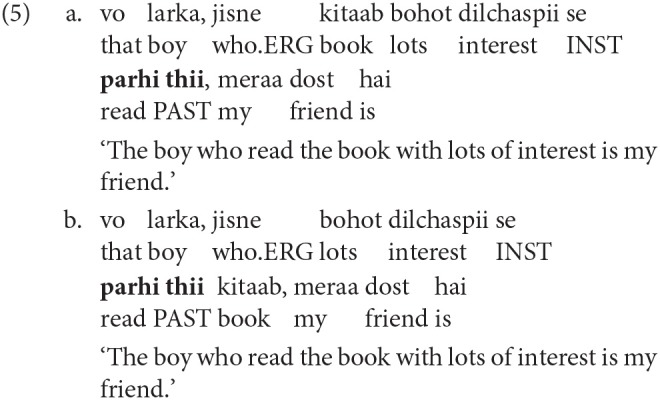


Online sentence comprehension in SOV languages is known to be incremental (Frazier, 1987) and predictive. Such a top-down parsing process involves establishing various syntactic relations based on the linguistic cues of a sentence and (as discussed above) the role of preverbal case-markers in achieving this is known to be critical. Given the evidence of syntactic priming during comprehension in these languages, the predictive aspect of the comprehension process and its effect on syntactic priming becomes important. Here, we want to explore how the use of prime structure and preverbal linguistic cues (i.e., case markers) of the target sentence interact during the comprehension of the target sentence. While several constraints on syntactic priming have been investigated, e.g., influence of lexical overlap, frequency of prime structure, etc., the interaction between the prime structure and the syntactic properties (in terms of preverbal case-marker) of the target during online comprehension has so far not been studied. Such an investigation will shed light on priming processes (and more generally sentence comprehension) in SOV languages.

In this work we investigate this interaction by manipulating the probability of using the prime structure while parsing the target sentence. This is done by using different case-markers in the target sentence. If priming during comprehension is influenced by the structural compatibility of the prime and target then we expect to observe an attenuation in the strength of priming when the integration of the target case-marker with the prime structure leads to a rare syntactic parse. On the other hand, if the integration leads to a frequent parse, then the strength of priming increases. We call this the Prime-Target Structural Compatibility Hypothesis: ‘During comprehension, syntactic priming is stronger when the linguistic cues of the target and the prime structure combine to form a frequent structure, otherwise priming effect is weak.' During online comprehension, the prime-target structural compatibility hypothesis, can be operationalized using the surprisal metric. In cases of high surprisal in the target sentence, we would expect to see no facilitation at the critical verb, in spite of the structural/lexical overlap between the prime and target. In fact, in such cases, a target structure with low surprisal (and a prime-target mismatch) should be processed faster. The prediction of the prime-target structural compatibility hypothesis with regard to online comprehension can be pitted against the Residual Activation Theory. If the residual activation theory is correct then we should find no effect of such a prime-target compatibility, especially when the verbal heads of the prime and target are identical. However, if the prime-target structural compatibility hypothesis is correct then such the prime-target compatibility should matter and would suggest a constraint on syntactic priming during comprehension of SOV languages.

The paper is arranged as follows: We first discuss some salient characteristics of Hindi word order and case system. Following this, we present a norming study that provides us with the material used in the critical experiments. We then discuss a sentence completion study to investigate the effect of preverbal case-marked nouns on priming. Next, we discuss a self-paced reading experiment to demonstrate the influence of case-markers on priming during online comprehension. Finally, we discuss the implications of our results.

## 2. Hindi Word Order and Case-System

Hindi is an Indo-European language spoken in India. At 615 million speakers, it is the third most widely used language in the world^1^. Hindi has a default SOV word order but allows word order flexibility. Consequently, it also has case-markers, a relatively rich morphology with verb-subject, noun-adjective agreement. Examples 6b-e below show some of the possible word order variations possible with 6a. These permutations are not exhaustive–in fact all 4! permutations are possible. Verbal arguments and adjuncts can be marked with case-markers. For example in 6a, the subject (*abhay* ‘Abhay') has an Ergative case-marker (*ne*), the indirect object (*kavitaa* ‘Kavita') has a Dative case-marker (*ko*). The object (*ek phuul* ‘a flower') is unmarked and the verb agrees with it. In general, the verb agrees with the highest unmarked argument.


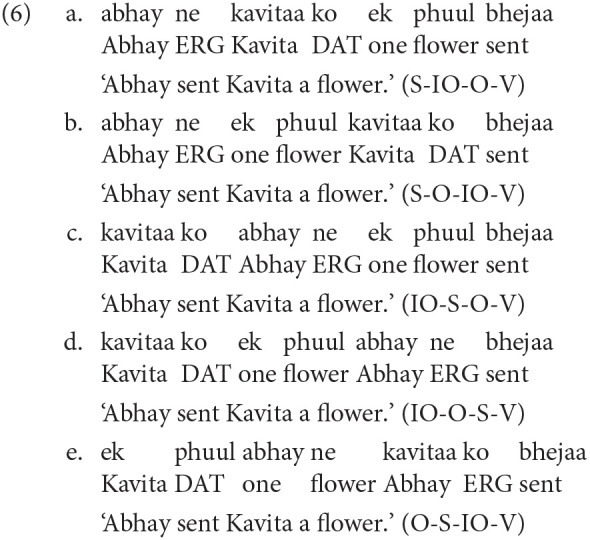


The language allows for various non-finite verbal forms that carry different inflections, for example *kara* (encodes causality/sequentiality), *e (hii)* (sequentiality), *taa huaa* (signifies co-occurring event), *naa* (gerundive form), etc. Some of these form can take their own argument structure, for example in 7 the noun-finite verb (*khaane* ‘eat') has its subject (*raam* ‘Ram') and its object (*khaanaa* ‘food'). In such cases, the subject is marked with a Genitive case-marker (here *ke*). The Genitive case-marker can also be marked to encode possession with a nominal head. For more details on various aspect of Hindi grammar, see (Kachru, 2006).


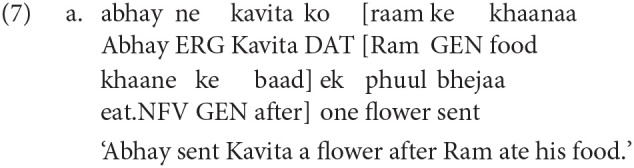


## 3. Experiment 1

Before discussing the critical experiments, we first discuss a norming study that demonstrates that a Hindi non-finite verb (NFV) structure is sensitive to structural priming. As stated in the previous section, Hindi allows two distinct usages of the Genitive case-marker, (i) as a marker to encode possession (e.g., *abhay kaa ghar* ‘Abhay's home') as shown in 8a, and (ii) to mark the subject of a non-finite verb, for example, in 8b *abhay kaa* ‘Abhay GEN' is the subject of the non-finite verb *jaanaa* ‘going.' The items in the studies discussed in Experiments 2 and 3 use the Genitive case in this latter role. The norming study was meant to demonstrate that Hindi native speakers can be primed for such a non-finite structure where the Genitive marks the subject of the non-finite verb. Demonstrating this is essential as the Genitive as possession usage is much more common in Hindi.


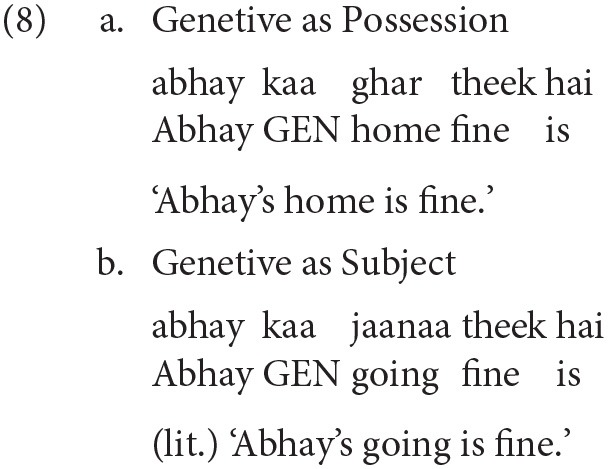


### 3.1. Materials and Methods

#### 3.1.1. Participants

28 native speakers of Hindi participated in this experiment^2^. The average age of the participants was 21.58 (SD: 3.01). Each participant was paid INR 200 for participating in the experiment.

#### 3.1.2. Items

Examples (9a) and (9b) show the experimental items. In the Non-finite Prime condition (a), the Genitive case-marked noun in the prime is the subject of the intransitive non-finite verb *hasnaa* ‘laughing.' In the Noun Prime condition (b), the Genitive case-marker acts as a possessor marker for the proper noun *raam* ‘Ram.' The target string (*mohan kaa* ‘Mohan=GEN') in both the conditions is identical. Twenty-four sets of items were prepared for all conditions. seventy-two filler items were used along with the experimental items. Compared to the critical items, fillers items comprised of different constructions and were designed based on the following criteria, (i) length of prime sentence and incomplete target sentence was kept similar to the length of the critical items, (ii) context in some fillers were generic questions, while the target had varying construction types, e.g., simple declarative with a transitive verb, (iii) some fillers had prime similar to the critical items but the target constructions differed, (iv) both prime and target differed from the critical items (see, 10). In addition, items for Experiment 2 also acted as fillers. A latin-square design was used to present different items. The complete list of experimental items can be obtained from the authors.


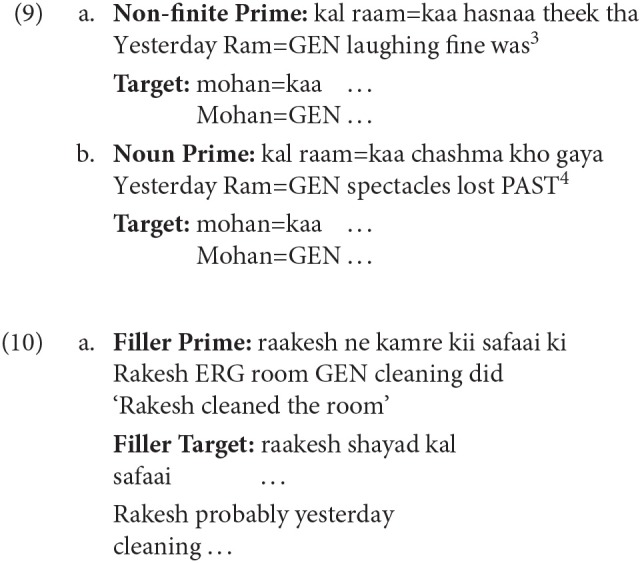


#### 3.1.3. Procedure

The sentence completion task was employed as the experimental paradigm (Taylor, 1953). The experiment began by explaining the task to the participants verbally as well as in written form. After this, several practice sentences were presented in order to familiarize participants with the task. Initially, a ‘+' sign appeared on the left of the computer screen signifying the start of a trial. When the space-bar button was pressed, the prime sentence was displayed. Pressing the space-bar button again displayed the incomplete target sentence. A …  symbol prompted the participant to complete the incomplete target sentence. This was done via a text box that appeared by pressing ‘space-bar' after the …  symbol. After typing in the text, the participants pressed the ‘enter' key to move on to the next trial. Participants were instructed to complete the incomplete sentence meaningfully. The experiment was conducted using Douglas Rohde's Linger software, version 2.94. Items were automatically randomized by Linger.

### 3.2. Response Coding

Each participant's responses obtained from the completion task were coded for two information, (1) presence of a non-finite verb such that the Genitive is the subject of the verb, and (2) presence of a noun such that the Genitive is related to the noun. With regard to non-finite verb completions, only intransitive verbs were deemed correct as the Genitive-as-Subject prime has an intransitive non-finite verb (see, example 9a). For the Non-finite prime condition and the Noun prime condition, 22.8 and 7.8% completions respectively did not belong to either of the two classes.

### 3.3. Prediction

The residual activation theory (Pickering and Branigan, 1998; Tooley and Traxler, 2010; Arai, 2012) that assumes linguistic heads (e.g., verbs) to encode syntactic information regarding their complements (e.g., arguments) would predict the prime sentence to influence the completion of the incomplete target sentence. In particular, it is expected that, in the Non-finite Prime condition (i.e., condition a), the Genitive case in the target should be treated as a subject and consequently more intransitive non-finite verbs should be used to complete the target in this condition. However, the Genitive case in the Noun Prime condition (i.e., condition b) should be treated as signifying possession which should lead to increased nominal completions in this condition. Conversely, we expect less use of nouns in the Non-finite Prime condition, and less use of intransitive non-finite verbs in the Noun Prime condition.

### 3.4. Results

All statistical analyses have been done using the generalized linear mixed-effects model. In particular, completion response analysis was done using the generalized linear mixed-effects model with logit link function. This has been done using the lme4 package (Bates et al., 2015) in R. The dependent variable was either the number of noun completions or the number of NFV completions. The predictor variable was the experimental condition (Noun-finite Prime vs. Noun Prime). This was dummy coded with a treatment contrast—the Non-finite Prime condition was coded 0 (and was therefore acted as the baseline) and Noun Prime was coded 1. The model selection was done based on the procedure in Matuschek et al. (2017). Likelihood ratio test (LRT) was used to compare a complex model with a simpler one. Five models were compared to each other. These were (1) a maximal model containing both subject and item-based random slopes, (2) a maximal model with no correlation parameter for the item/subject component, (3) a reduced model with only subject specific random slope, (4) a reduced model with only item specific random slope, and (5) a reduced model with no random slopes. For the non-finite completion as well as the noun completion models, the LRT tests selected the model with only subject specific random slopes, i.e., model 3.

Results show that in the Non-finite Prime condition, the number of intransitive non-finite verb completions was more compared to the Noun Prime condition (z = −4.78). In the Noun Prime condition, the number of noun completions was more than the Non-finite Prime condition (z = 6.12). This can be clearly seen in Tables 1, 2. The results also show that the number of noun completions were relatively more in the condition with a non-finite prime (condition a), compared to the non-finite completions in the condition with noun prime (condition b). Example 11 shows a representative completion where the priming in the respective conditions successfully takes place.


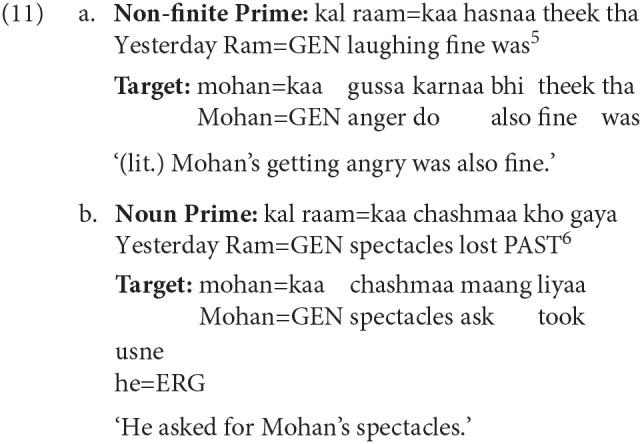


**Table 1:** Percentage completions of intransitive non-finite verbs and nouns in conditions (a) and (b) for Experiment 1. We only consider intransitive non-finite verb as correct completions.

**Condition**	**Non-finite verb completions**	**Noun completions**
Non-finite Prime	42.5%	34.7%
Noun Prime	8.5%	83.7%

**Table 2:** Glmer results for the non-finite verb and noun completions in the two conditions for Experiment 1. The analysis uses treatment contrast, with the Non-finite Prime condition as the baseline with the coefficient signifying the difference from the baseline.

	**Non-finite verb**				**Noun**		
	**Coefficient**	**SE**	**z-value**		**Coefficient**	**SE**	**z-value**
Intercept	–0.39	0.25	–1.55		–0.89	0.32	–2.74
Noun Prime	–2.52	0.52	**–4.78**		3.27	0.53	**6.12**

*Statistically significant coefficients with t-value more than 2 appear in bold font*.

### 3.5. Discussion

The results show that it is possible to prime Hindi native speakers to use the Genitive case-marker differentially based on the syntactic structure of the prime sentence. The sentence completion results clearly show that when the prime has a non-finite clause with a Genitive subject, the number of completions with Genitive as a subject marker increases. This leads to an increased use of a intransitive non-finite verb in the Non-finite Prime condition compared to the Noun Prime condition. Conversely, when the prime sentence has a simple noun phrase with the Genitive possessor, the number of completions with Genitive as a possessive marker increase. This leads to an increased use of the noun phrase in the Noun Prime condition compared to the Non-finite Prime condition. These results are consistent with the predictions of the residual activation theory (Pickering and Branigan, 1998). In addition, the study demonstrates that a rare syntactic structure (here, a non-finite clause with a Genitive marked subject) can be primed successfully in an SOV language like Hindi.

The results also show that the number of Genitive as possessor occurrences were quite high in the Non-finite Prime condition in spite of the presence of non-finite prime. Compared to this, the number of Genitive as subject marker occurrences in the Noun Prime condition was quite low. This is not surprising as this reflects the default use of the Genitive case-marker. Also, the relative increase in the non-finite verb vs noun phrase completions across the two conditions was different. The percentage non-finite completion in the Noun Prime condition was 8.5 and 42.5% in the Non-finite Prime condition—a difference of 34%. The percentage noun phrase completions in the Non-finite Prime condition was 34 and 83.7% in the Noun Prime condition—a difference of 49%.

The norming study provides a useful baseline for the experiments presented in the next two sections. It demonstrates that the Genitive case-marker can be primed in its marked role (that of subject of a non-finite verb) when an appropriate prime sentence is available. We will use this finding in the design of the experiment discussed next.

## 4. Experiment 2

As stated in the Introduction section, both syntactic priming as well as the use of preverbal case-markers are known to influence processing in SOV languages. However, the interaction between the prime structure and the preverbal linguistic cues in the target sentence has not been investigated rigorously. The aim of the current experiment was to, therefore, investigate this interaction. This is done by keeping the prime sentence constant and varying the probability of using the prime structure while parsing the target sentence. We do this by manipulating preverbal case-markers in the target sentence of various conditions.

### 4.1. Materials and Methods

#### 4.1.1. Participants

27 native speakers of Hindi participated in this experiment. The average age of the participants was 21.56 (SD: 2.9). Each participant was paid INR 200 for participating in the experiment.

#### 4.1.2. Items

We first illustrate the items using a template shown below.


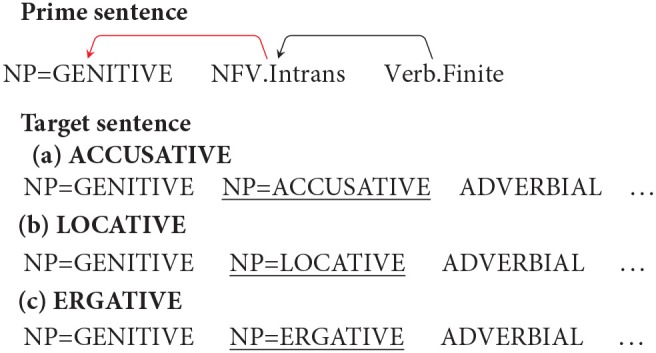


Similar to the norming study, each target item is preceded by a prime that had a non-finite clause with an intransitive non-finite verb (NFV.Intrans). The Genitive marked noun acts as a subject of the NFV.Intrans in each condition. The target in the Locative condition had a Genitive marked noun followed by an NP with a Locative (LOC) case. The target in the Accusative and Ergative conditions had a Genitive marked noun followed by an NP with an Accusative (ACC) and an Ergative (ERG) case respectively. In all the three conditions, the case-marked noun was followed by an Adverbial. As stated above, the use of different case-markers was meant to manipulate the probability of using the prime structure in completing the target sentence. In particular, in the Locative condition the probability of using the prime structure in the target is high compared to the probability of using the prime structure in the Accusative and Ergative conditions. This is because using the prime structure in the target for the Locative condition will lead to a non-crossing structure, while in the Accusative and Ergative conditions, use of prime will lead to a crossing dependency. This is clearly shown in Table 3. The conditions with Accusative and Ergative case lead to a crossing structure as these case-markers cannot be grammatically integrated with the intransitive non-finite verb, rather they need to be syntactically associated with an appropriate matrix verb (appearing after the NFV). Structures with crossing dependencies are known to be rare cross-linguistically (Nivre and Nilsson, 2005; Havelka, 2007; Ferrer-i-Cancho et al., 2018). In a Hindi dependency treebank (Bhatt et al., 2009), only 1% of the total 6,342 non-finite clause instances were found to have crossing dependencies. Note that it is possible to posit a non-finite verb in the Accusative condition without forming a crossing dependency. However, in that case the non-finite verb would have to be Transitive (rather than the intransitive verb used in the prime sentence)^7^.

**Table 3:** Illustration of structures for the target sentence as predicted by the residual activation theory for conditions in Experiment 2. If priming happens, then the critical NP=GEN should be treated as the subject of the NFV.Intrans (shown as a red arc above). In order to use the prime structure in the target for Accusative and Ergative conditions, a crossing dependency (shown as a “dashed” arc above) has to be posited.

**Prime structure**	**Expected structure in the target assuming successful priming**
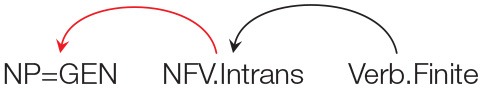
	**Condition Accusative: NP=GEN NP=ACC ADV …** 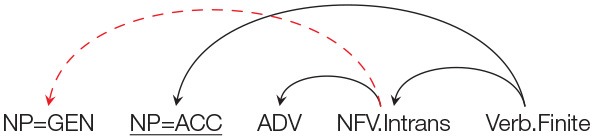
	**Condition Locative: NP=GEN NP=LOC ADV …** 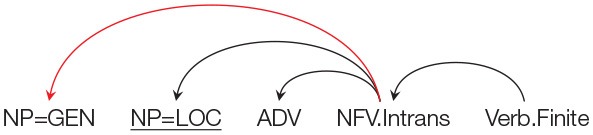
	**Condition Ergative: NP=GEN NP=ERG ADV …** 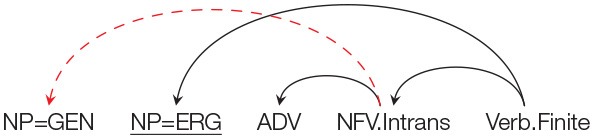

Example 12 shows a sample experimental item. The target items in the three conditions were determined by the case of an NP (*abhay* in examples 12a–12c). The case-marker on the noun (*abhay*) in the Accusative, Locative and Ergative conditions was Accusative, Locative and Ergative respectively. Each target item is preceded by a prime that had a non-finite clause with an intransitive non-finite verb (NFV). The prime sentence was identical for all the conditions. In the example below, this NFV clause corresponds to *kal raam=kaa hasnaa* ‘yesterday Ram's laughing' (literal translation). The NP=GENITIVE (*raam=kaa* ‘Ram's') in the prime acts as the subject of the intransitive NFV (*hasnaa* ‘laughing'). Target items were presented until the adverbial (*bevajah* ‘unnecessarily') during the completion task.


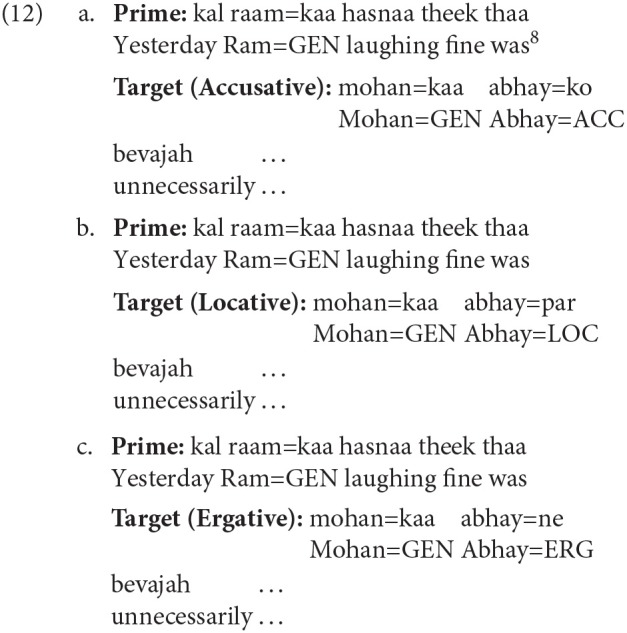


Twenty-four sets of items were prepared for all conditions. Seventy-two filler items were used along with the experimental items. Since this experiment was run along with the norming study, the filler items was similar to the ones discussed in section 3.1.2. A latin-square design was used to present different items. The complete list of experimental items can be obtained from the authors.

#### 4.1.3. Procedure

Similar to experiment 1, we follow the sentence completion paradigm. For details, see the Procedure section for Experiment 1.

#### 4.1.4. A Note on the Experimental Design

A careful reader would have noticed that the above experimental design only uses prime conditions (which have identical prime sentences). It does not have any conditions without the prime, which is typically the case in the priming literature. This was a conscious decision. Our main goal in this experiment was to understand the differential effect of case-marker on priming; in that sense we are interested in the RELATIVE effect of differing case-markers on priming strength, rather than the ABSOLUTE effect of priming vs. no priming. Experiment 1 has already demonstrated that priming a rare participle structure is possible in Hindi. In the light of Experiment 1, the question that we ask in this experiment is: how much of this priming is influenced by the presence of different preverbal case-markers in the target sentence? The other reason for not including conditions without primes was that the presence of a marked construction in the priming condition could bias the completions in the conditions with no prime.

### 4.2. Response Coding

Similar to the previous experiment, participants' responses obtained from the completion task were coded for two information, (a) presence of an intransitive non-finite verb such that the Genitive marked NP is the subject of the non-finite verb, and (b) presence of a noun such that the Genitive marked NP is related to the noun. For the Accusative case condition, 76.6% of completions did not belong to either of the two classes. This percentage for the Locative and Ergative conditions was 3.8 and 0.8% respectively.

### 4.3. Prediction

The results of the norming study show that given the appropriate prime sentence, the Genitive marked NP in the target sentence can be primed to act as a subject of a intransitive non-finite verb (NFV.Intrans). For the current experiment, the residual activation theory (Pickering and Branigan, 1998) would therefore predict that participants should complete the target sentence in conditions (12a), (12b), and (12c) with a non-finite clause and an intransitive NFV, i.e., similar to the prime structure, participants should use the Genitive case in the target sentence as the subject of the intransitive NFV. The NFV should, of course, be followed by some matrix verb in order for the completion to be grammatical. The structures of these completions can be seen in Table 3. If no priming happens then NP=GEN should be completed as part of a noun phrase signifying possession and no intransitive NFV should be used. Given that the three conditions can be grammatically completed using an intransitive NFV, the residual activation theory (Pickering and Branigan, 1998) will therefore predict no difference in the number of intransitive NFV completions across the three conditions.

However, if the Prime-Target Structural Compatibility Hypothesis^9^ is correct then syntactic priming will be influenced by the probability of using the prime structure while parsing the target sentence. In such a scenario, the priming effect should be strongest in the Locative condition; while the priming effect should be weak in Accusative and Ergative conditions. As stated earlier, this is because, the probability of using the prime structure in the target sentence in Accusative and Ergative conditions, is low compared to the probability of using the prime structure in the Locative condition. In particular, while the NP=LOC can be structurally integrated with the primed intransitive NFV, an NP=ACC or an NP=ERG cannot be integrated. This is because an intransitive NFV in Hindi cannot take an ACC/ERG marked argument. In order to posit an intransitive NFV in the ACC/ERG condition, the NP=ACC/ERG needs to be integrated with the matrix verb (that should appear *after* the NFV clause) by making a discontiguous phrase (see Table 3)^10^. Such crossing dependencies are rare in Hindi^11^. Table 4 summarizes the predictions of the two theories with regard to the expected syntactic priming in the three conditions.

**Table 4:** Predictions of the Residual activation theory (RA) and the Prime-Target Structural Compatibility Hypothesis (PTSC) for the syntactic priming effect in the three conditions for Experiment 2.

**Condition**	**RA**	**PTSC**
Accusative	Yes	No/Less
Locative	Yes	Yes/More
Ergative	Yes	No/Less

### 4.4. Results

All statistical analyses have been done using the generalized linear mixed-effects model using the method discussed in Experiment 1. Likelihood ratio test (LRT) was used to compare a complex model with a simpler one. Five models were compared to each other. These were (1) a maximal model containing both subject and item-based random slopes, (2) a maximal model with no correlation parameter for the item/subject component, (3) a reduced model with only subject specific random slope, (4) a reduced model with only item specific random slope, and (5) a reduced model with no random slopes. For the intransitive non-finite completion model, the LRT tests selected the model with only subject specific random slopes, i.e., model 3. For the noun completion model, model 3 got selected. For identical intransitive non-finite verb completion model, model 5 got selected.

Results show that compared to the Locative condition, the number of intransitive non-finite verb completions was less in the Accusative condition (z = −3.48) and also in the Ergative condition (z = −5.01). There was no difference in such completions between Accusative and Ergative conditions (z = −0.89). For noun completions, compared to the Locative condition, the number of such completions was less in the Accusative condition (z = −2.14) and more in the Ergative condition (z = 6.56). The completion percentages are shown in Table 5. The glmer results are shown in Tables 6, 7.

**Table 5:** Percentage completions of intransitive non-finite verbs and nouns in Accusative, Locative, and Ergative conditions for Experiment 2.

**Condition**	**Intransitive non-finite verb**	**Lexically identical intransitive non-finite verb**	**Noun**
Accusative	10%	0.67%	13.4%
Locative	56%	10.9%	40.2%
Ergative	7.7%	2.8%	91.5%

**Table 6:** Glmer results for the non-finite verb and noun completions Experiment 2. The analysis uses treatment contrast, with the Locative condition as the baseline with the coefficient signifying the difference from the baseline.

	**Intransitive non-finite verb**				**Noun**		
	**Coefficient**	**SE**	**z-value**		**Coefficient**	**SE**	**z-value**
Intercept	0.28	0.21	1.28		–0.39	0.32	–1.22
Accusative	–3.48	0.77	**–4.48**		–2.14	0.45	**–4.76**
Ergative	–5.01	1.52	**–3.28**		6.56	2.30	**2.85**

*Statistically significant coefficients with t-value more than 2 appear in bold font*.

**Table 7:** Glmer results for the (Identical) Intransitive Non-finite verb in the three conditions for Experiment 2. The analysis uses treatment contrast, with the Locative condition as the baseline with the coefficient signifying the difference from the baseline.

	**(Lexically identical) Intransitive non-finite verb**		
	**Coefficient**	**SE**	**z-value**
Intercept	–2.66	0.51	–5.16
Accusative	–3.10	1.05	**–2.94**
Ergative	–1.70	0.60	**–2.80**

*Statistically significant coefficients with t-value more than 2 appear in bold font*.

The completion results also show that the number of intransitive non-finite verb completions that were lexically identical to the prime non-finite verb was more in the Locative condition compared to the Accusative condition (z = −2.94) and the Ergative condition (z = −2.80). Example 13 shows a representative completion in the three conditions. The non-finite verb used in the Accusative condition is Transitive, while in the Locative case the non-finite verb is intransitive. In the Ergative condition, no non-finite verb is used.


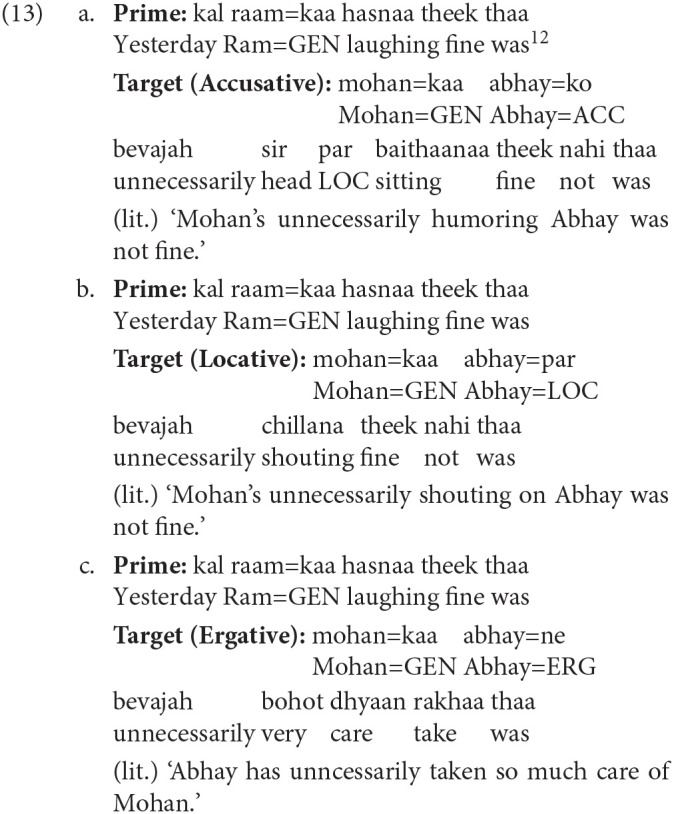


A careful reader would have noticed that while the completions percentages in Table 4 for Locative and Ergative conditions sum up to approximately 100%, this percentage for the Accusative condition was around 23%. This is because in this condition around 76% of completions had *transitive* non-finite verbs^13^. Recall that the prime had an *intransitive* non-finite verb. Given the assumptions of the residual activation theory (Pickering and Branigan, 1998), an *intransitive* non-finite verb in the prime sentence should not lead to the usage of a *transitive* non-finite verb in the target sentence. This is because the theory assumes transitive combinatorial nodes to be connected to verb lemma nodes that are transitives; the transitive combinatorial nodes are not connected to the intransitive verb lemma nodes. In order to investigate if such a high percentage of transitive non-finite verb in the Accusative condition was caused due to the prime structure, we ran a pilot completion study using the items in Experiment 2.

### 4.5. Experiment 2a: Pilot Study

Compared to Experiment 2, the only difference in the pilot study (*N* = 15) was that the target sentences were not preceded by the appropriate prime. Instead, they were preceded by a question ‘What happened?.' The sample item is shown in (14). This study followed the same procedure as Experiment 2 and had items such as 14a-c below. The average age of the participants was 22.07 (SD: 3.45).


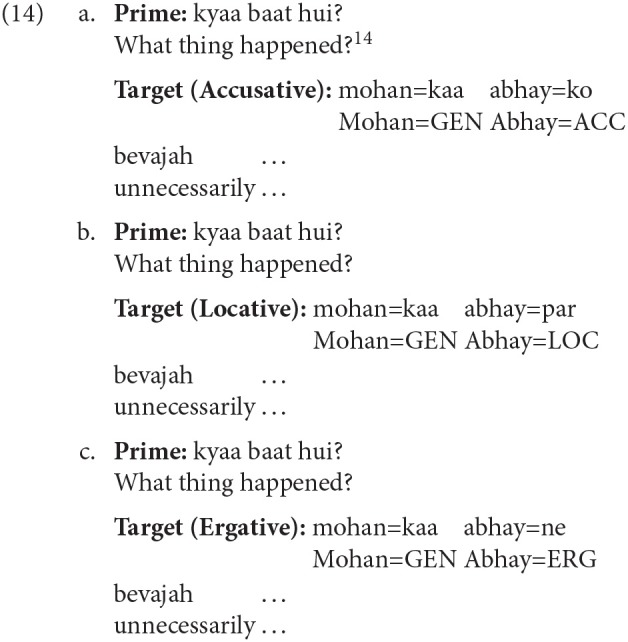


Results for the pilot completion study shows that in Accusative and Locative conditions, noun completions were more (see Table 8) compared to the completion study where the NFV prime was present (Experiment 2 discussed earlier). Not much difference was seen in the Ergative condition. Further, results show that the total number of transitive non-finite verb completions was already high (57%) in the Accusative condition when the intransitive NFV prime was not present (see Table 8). This suggests that, in this condition, the Accusative case-marker can trigger a transitive non-finite completion independent of the prime. However, as stated earlier, when the intransitive NFV prime was present, the percentage of *transitive* NFV completion was around 76%. This suggests that the presence of an intransitive non-finite prime does increase the completions for transitive non-finite structure in the target for the Accusative condition. This priming effect across verb class is quite interesting and is hard to reconcile with the assumption of linguistic representations made by the residual activation model (Pickering and Branigan, 1998). Particularly, according to the residual activation model, priming is triggered by the combinatorial property of the verb. The results of the pilot study points to a mechanism of priming that is non-lexical in nature and could be compatible with recent finding on graded predictability (Staub, 2015; Luke and Christianson, 2016). Given the preliminary nature of the pilot study, further investigations will be required to confirm these findings. We discuss this point further in the General Discussion section.

**Table 8:** Percentage completions of non-finite verbs and nouns in Accusative, Locative and Ergative conditions with no NFV prime in the pilot study (Experiment 2a).

**Condition**	**Non-finite verb completions (intransitive)**	**Non-finite verb completions (transitive)**	**Noun completions**
Accusative	21.78%	57.42%	20.7%
Locative	39.81%	0.92%	59.2%
Ergative	9.34%	0%	90.6%

### 4.6. Discussion

Experiment 2 results show that syntactic priming of an intransitive non-finite verb is affected by the probability of using the prime structure in the target sequence. When this probability was very low, the expected priming effect was not very strong. In particular, we found that the effect of syntactic priming was most pronounced in Locative case condition compared to the Accusative and Ergative case conditions. The results go against the current formulation of the residual activation theory and support the Prime-Target Structural Compatibility Hypothesis.

Syntactic priming in a language like Hindi is being modulated by the presence of case-markers in the target sentence. Indeed, different case-markers (ACC, LOC, ERG) modulated the probability of using the prime structure while parsing the target sentence. Recall that it is grammatically possible to use an intransitive non-finite verb in Accusative and Ergative conditions. However, doing so, would create a crossing dependency (see Table 3). This is because an intransitive verb cannot take an Accusative or an Ergative case-marked noun as its argument. The noun with the Accusative/Ergative case-maker will need to attach to a matrix verb after the non-finite verb, making it a rare structure. The results therefore suggest that for such configurations, priming is being partly overridden by a constraint of the parser to avoid building a rare target structure.

Can the results of Experiment 2 be explained by the difference in the transition probability of NP-ACC/NP-ERG followed by NP-GEN? In that case, the results can be frequency related rather than due to syntactic priming^15^. The pilot study (Experiment 2a) shows that in the Locative and Accusative conditions, noun completions were higher compared to the completion study where the NFV prime was present. Since the transition probabilities between NP-GEN NP-LOC/ACC will be identical between these two completion studies, the completion results should be expected to be identical. However, this is not the case. This shows that the presence of the prior prime sentence in Experiment 2 is influencing the completions and transition probabilities alone cannot explain the results.

Together the two completion studies (Experiment 2 and 2a) suggest that, while building a syntactic structure of the target sentence, the top-down parsing process continuously evaluates the prime structure in light of the linguistic cues in the target sentence. If using the prime structure in the target leads to a rare syntactic configuration, then the parser can override the prime structure in favor of a more frequent structure. Otherwise, the prime structure is incorporated with the syntactic information of the target sentence.

Additionally, vis-à-vis the SPR experiment that we discuss next, we performed the Experiment 2 completion study in order to be certain regarding the prediction of the critical verb in each experimental items. The sentence completion task (Taylor, 1953) is the go-to paradigm to quantify predictability during comprehension. Previous studies on word predictability employing the sentence completion task have shown that completion patterns correlate strongly with reading time patterns found during online sentence comprehension (Rayner et al., 2011; Levy and Keller, 2013; Husain et al., 2014; Jäger et al., 2015). Since, the sentence completion task is known to provide the most comprehensive measure to quantify predictability of upcoming material during online comprehension (Staub, 2015), the pattern of the current completion study will predict that in conditions where priming is weak, there should be processing difficulty if the participants read the target with the expected prime structure. We test this in the next experiment.

## 5. Experiment 3

The aim of the current experiment was to investigate the effect of syntactic priming during online sentence comprehension in a language like Hindi. This experiment is informed by the results obtained in the completion study discussed in Experiment 2.

### 5.1. Materials and Methods

#### 5.1.1. Participants

75 native speakers of Hindi participated in this experiment. The average age of the participants was 22.36 (SD: 3.2). Each participant was paid INR 100 for participating in the experiment. The subjects in the current study did not participate in other experiments discussed in this paper.

#### 5.1.2. Items

Items from experiment 2 were used. The target items had the same intransitive non-finite verb (e.g., *hasnaa* ‘laughing') in all the three conditions. The non-finite verb was the critical region in all conditions. // below signify regions in the Self-paced reading (SPR) experiment.


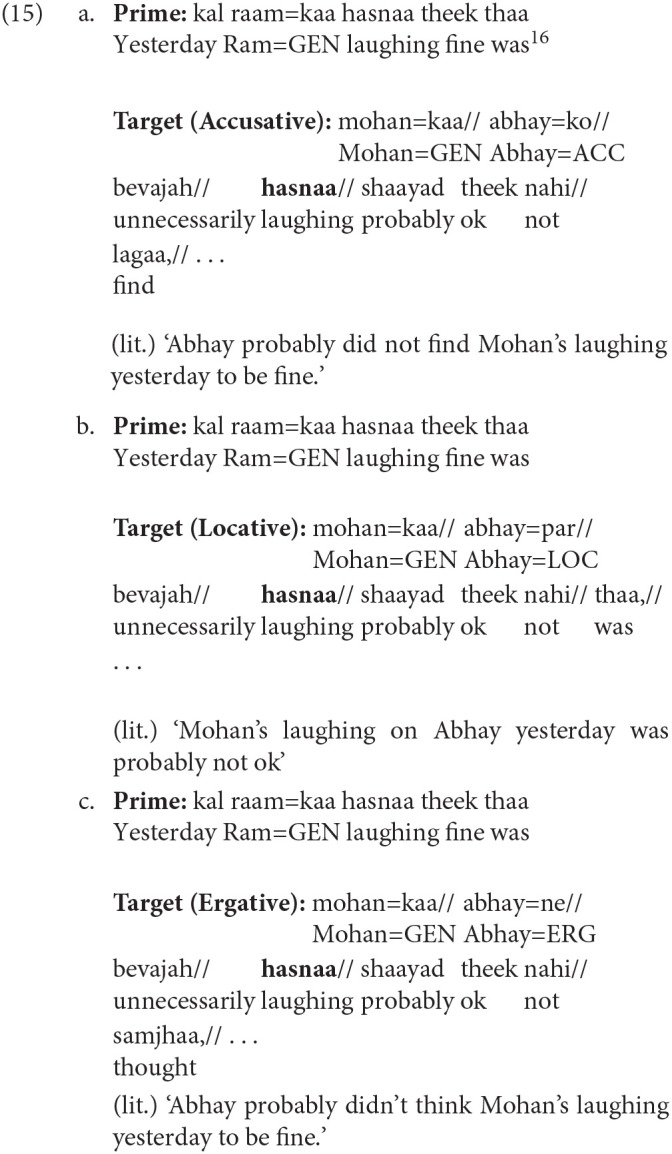


Twenty-four sets of items were prepared for all conditions. Seventy-two filler items were used along with the experimental items. A latin-square design was used to present different items. The complete list of experimental items can be obtained from the authors.

#### 5.1.3. Procedure

We used the moving window self-paced reading paradigm (Just et al., 1982) for the reading task. Stimulus items were presented using Douglas Rohde's Linger software, version 2.94. A Latin square design ensures that each participant sees each item in only one condition. The target items and fillers were randomized for each participant.

The experiment began by explaining the task to the participants verbally as well as in written form. After this, several practice sentences were presented in order to familiarize participants with the task. At the beginning of each trial, a ‘+' sign appeared on the left of the computer screen. When the participant pressed the space-bar key this + sign got replaced with the prime sentence. Pressing the space-bar again displayed the words in the target sentence as dashed lines. With each successive press of the space bar, each word or phrase was unmasked while masking the previously seen word in a moving window fashion. This successive replacement continued until the participant had read the whole sentence. Reading times or RTs (in milliseconds) were taken as the measure of relative momentary processing difficulty. In around 75% of trials yes-no comprehension questions were shown. The questions varied in their complexity and probed comprehension from various parts of the sentence to ensure that participants did not build any strategy. The number of questions with correct ‘yes' responses were equal to the correct ‘no' responses. The f-key was pressed for answering a question with a ‘yes' response and the j-key will be pressed for answering with a ‘no' response.

### 5.2. Predictions

Based on the completion results in experiment 2, we predict reading time at the critical non-finite verb in the Locative condition to be faster than Accusative and Ergative conditions. This is because, the participants should expect to see the primed non-finite verb in the target sentence in the Locative condition. However, in the Accusative condition they expect to see a verb of a different verb class and in the Ergative condition, they rarely expect to not see a non-finite verb. In effect, we expect that, during online comprehension, the predictions of the Prime-Target Compatibility Hypothesis regarding syntactic priming will be correct and the predictions of the Residual activation theory will be incorrect. As stated in the introduction, for online comprehension, the Prime-Target Compatibility Hypothesis can be operationalized using the surprisal metric. Since the SPR paradigm is known to have spillover effects (Kaiser, 2014), the predicted RT difference can also appear at the post-critical region. Consequently, similar to the critical region, the immediate post-critical region in all the conditions was kept identical.

### 5.3. Results

All statistical analyses have been done using the generalized linear mixed-effects model. Question response analysis was done using generalized linear mixed-effects with logit link function. This was done using the lme4 package (Bates et al., 2015) in R. The model selection was done based on the procedure in Matuschek et al. (2017). Likelihood ratio test (LRT) was used to compare a complex model with a simpler one. Five models were compared to each other. These were (1) a maximal model containing both subject and item-based random slopes, (2) a maximal model with no correlation parameter for the item/subject component, (3) a reduced model with only subject specific random slope, (4) a reduced model with only item specific random slope, and (5) a reduced model with no random slopes. For the critical region as well as the post-critical region, the LRT tests selected the maximal model, i.e., model 1. Raw RT were log transformed before fitting the lmer models. All comparisons were done by treating the Locative condition as the baseline condition.

Question response analysis showed no difference in the comprehension accuracy when the baseline condition was compared to the Accusative condition (z = −0.45) and the Ergative condition (z = −0.35). Average comprehension accuracy for Accusative, Locative and Ergative conditions was 83, 84, and 82%, respectively. Mean comprehension accuracy across the three conditions was 83%.

**Figure 1:**
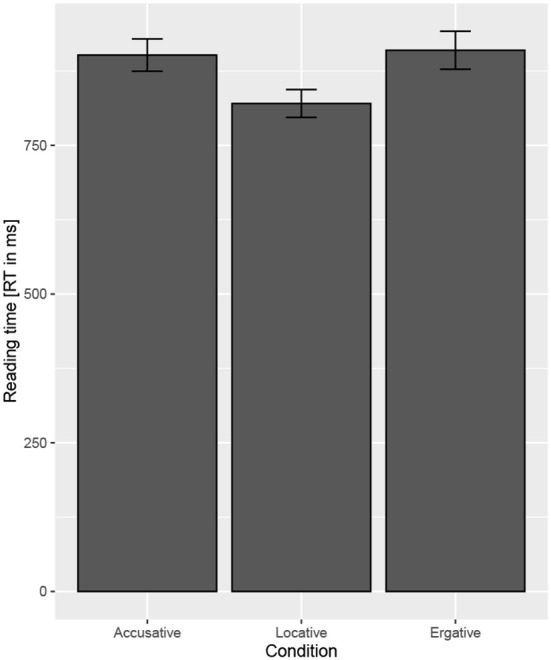
Reading time at the post-critical region for the three conditions in Experiment 3.

**Figure 2:**
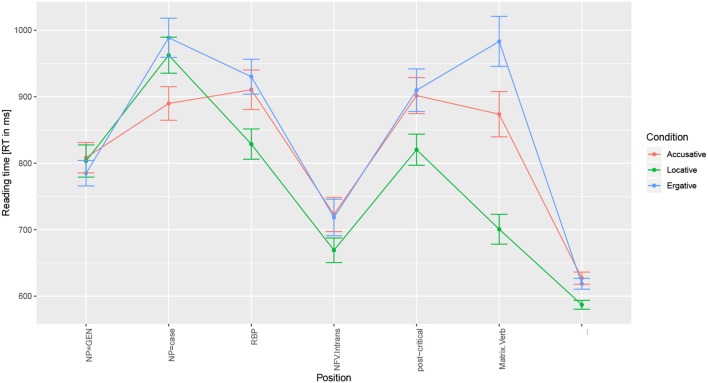
Reading time for all the regions in the target sentence for the three conditions in Experiment 3. “NFV.Intrans” is the critical region; “post-critical” is the post-critical region.

RT result at the critical region showed no difference when the baseline condition was compared to the Accusative condition (*t* = 0.87) and the Ergative condition (*t* = 1.02). RT result at the post-critical region (one region after the critical region which was identical in all the three conditions) showed a significant difference when the baseline condition was compared to the Accusative condition (*t* = 2.09) and the Ergative condition (*t* = 2.45) such that reading time was found to be faster in the Locative condition compared to the RT in the Accusative/Ergative condition. Table 9 shows the *t*-values, SEs, and the coefficient for the models. The RT difference between the three conditions at the post-critical region is shown in Figure 1, while Figure 2 shows the RT for each region in the target sentence for the three conditions. Interestingly, we find that RT is faster in the Locative condition compared to the Ergative condition (*t* = 3.7) at the adverbial (RBP) (see Figure 2); this could suggest that encountering an adverbial after an ERG case-marker is less expected in this configuration compared to encountering an adverbial after a LOC case-marker. In addition, we also find that the Locative condition is slower than the Accusative condition (z = −2.83) at the NP-case regions; comparison between conditions at this region is difficult to make due to differing lexical items. Similarly, a comparison at the Matrix.Verb region across the three conditions is difficult due to differing lexical items.

**Table 9:** Lmer results for reading time at the critical (non-finite verb) and the post-critical region for Experiment 3. The analysis uses treatment contrast, with the Locative condition as the baseline with the coefficient signifying the difference from the baseline.

	**Critical region**			**Post-critical region**		
	**Coefficient**	**SE**	***t*-value**	**Coefficient**	**SE**	***t*-value**
Intercept	6.38	0.03	181.39	6.55	0.04	136.66
Accusative	0.02	0.02	0.87	0.07	0.03	**2.09**
Ergative	0.03	0.03	1.02	0.07	0.03	**2.45**

*Statistically significant coefficients with t-value more than 2 appear in bold font*.

### 5.4. Discussion

The reading time results for the SPR study were as predicted and support the Prime Target Compatibility Hypothesis; reading time at the post-critical region in the Locative condition was lower than the reading time in Accusative and Ergative conditions. This pattern is consistent with the completion pattern found in Experiment 2. From a methodological perspective the results are consistent with Traxler and Tooley (2008) who found the SPR paradigm to be sensitive to priming effects.

Previous work on the effect of syntactic priming during comprehension (Tooley and Traxler, 2010) has consistently found that priming effects are robust when the lexical identity of the critical verb used in the prime and target is the same. The current experiment did ensure this. The results are therefore hard to reconcile with the residual activation-based account to syntactic priming. Since the critical verb in both the prime and the target are identical, it was expected that priming should lead to facilitation in all the three conditions. However, facilitatory effects were found only in the Locative condition. As noted, in Experiment 2, the Locative condition leads to the highest amount of priming effect in terms of the use of the correct non-finite verb. Combined with the results of the completion study, the SPR results shows that the priming effect is modulated by the linguistic cues (here, case-marker) in the target sentence during online comprehension. In particular, priming effect was found to be stronger when the syntactic structure of the prime and the linguistic cues of the target formed a frequent parse. When the parse was rare, syntactic priming suffered.

While the RT results cannot be explained by the residual activation account (Pickering and Branigan, 1998), it could be explained by the surprisal metric (Levy, 2008). The conditional probability of encountering an intransitive non-finite verb given previous ‘context' in Accusative and Ergative conditions is less than that in the Locative condition. It would therefore correctly predict a lower RT in the Locative condition compared to Accusative and Ergative conditions. As stated in the Introduction section, the surprisal metric can be used to operationalize the Prime-Target Compatibility hypothesis.

Recent results (e.g., Jaeger and Snider, 2013) suggest that priming effects can be equated to prediction error and that comprehenders act rationally to adapt their expectation based on their environment. One implication of such a proposal for our study would be that the processing cost in Accusative and Ergative conditions should reduce as the experiment progresses. In order to test this possibility, we conducted a *post-hoc* analysis by including trail id as a covariate in the linear mixed models discussed above. Any systematic reduction in processing cost due to trial progression in particular conditions should be seen as an interaction effect between the relevant condition and the trial id. Results are shown in Table 10. While we do find a significant effect of trial (*t* = −4.96), the results show no significant interactions. The significant effects found previously still persist in the new model.

**Table 10:** Lmer results for reading time at the critical (non-finite verb) and the post-critical region with trial id as a covariate for Experiment 3. The analysis uses treatment contrast, with the Locative condition as the baseline with the coefficient signifying the difference from the baseline.

	**Critical region**				**Post-critical region**		
	**Coefficient**	**SE**	***t*****-value**		**Coefficient**	**SE**	***t*****-value**
Intercept	6.38	0.03	184.44		6.55	0.04	138.11
Accusative	0.02	0.02	0.85		0.07	0.03	**2.19**
Ergative	0.03	0.03	0.95		0.07	0.03	**2.41**
Scale(trial id)	–0.01	0.002	–5.71		–0.01	0.002	**–4.96**
Scale(trial id):Accusative	0.004	0.003	1.24		–0.003	0.003	–0.96
Scale(trial id):Ergative	0.0005	0.003	0.17		–0.001	0.003	–0.34

*Statistically significant coefficients with t-value more than 2 appear in bold font*.

## 6. General Discussion

Together the completion and the SPR experiments show that syntactic priming during comprehension is modulated by the probability of using the prime structure in the target sentence. In addition, we found preliminary evidence that priming need not be solely driven by the combinatorial properties of a verb. These results go against the predictions of the Residual Activation Theory of syntactic priming.

### 6.1. Nature of the Parsing Process in SOV Languages

A key contribution of our work is that it highlights certain constraints on syntactic priming in an SOV language like Hindi. In particular, it shows that the strength of the priming effect is modulated by the compatibility of the prime structure with the preverbal linguistic cues (such as case-markers) in the target sentence. Previous work has highlighted certain typological differences in the way syntactic priming manifests in SOV languages such as Japanese vs an SVO language such as English (Arai, 2012). For example, it has been shown that syntactic priming in SOV languages can be triggered preverbally (Tanaka et al., 2007; Arai and Mazuka, 2010), also see Santesteban et al. (2015). While our work does not directly address this issue, it does suggest that preverbal nominal case-markers in the target sentence are an important determinant of syntactic priming during comprehension.

Table 11 shows the dominant completions for the various conditions across the three completion studies discussed earlier. We find that while building the syntactic structure of the target sentence, the top-down parsing system continuously evaluates the prime structure in light of the linguistic cues in the target sentence. In particular, the results suggest that syntactic priming is affected by the probability of using the prime structure while parsing the target sentence. If the integration of the prime structure and the linguistic cues (e.g., case-markers) in the target sentence results in a rare parse then the chances of utilizing the prime structure reduces. For instance, this was seen in Experiment 2 where the effect of priming was found in the form of higher use of non-finite structures in the completion of both Accusative and Locative conditions vs the Ergative condition. At the same time, Experiment 2a demonstrated the predictive effect of case-marker (in the absence of any priming) leads to more transitive non-finite verb completions in the Accusative condition, while for the Ergative condition there were more noun completions.

**Table 11:** Dominant completions with the Genetive case-marker across all the sentence completion studies for various conditions. NFV, non-finite verb; NFV.Trans, Transitive non-finite verb; NFV.Intrans, Intransitive non-finite verb; LOC, Locative case-marker; ACC, Accusative case-marker; ERG, Ergative case-marker.

	**No NFV prime**		**NFV.Intrans prime**	
	***No case-marker***	***Case-marker***	***No case-marker***	***Case-marker***
**Dominant**	Noun	NFV.Trans [ACC]	NFV.Intrans	NFV.Trans [ACC]
**completion**		Noun [LOC, ERG]		NFV.Intrans [LOC]
				Noun [ERG]

Given the pattern shown in Table 11, what can we say about the nature of the parsing system during sentence comprehension? We can hypothesize that (1) the parser avoids building structures that are rare, and (2) the parser uses inter-sentential structural information/cues (e.g., via priming) along with intra-sentential syntactic cues to build a parse. With regard to syntactic priming, these points are encapsulated in the Prime-Target Structural Compatibility Hypothesis. In this work, we operationalized rarity of a target parse via constructions that have crossing dependencies. The results, at the very least, show that syntactic priming is adversely affected in the case of certain complex structures, for example, the ones involving crossing dependencies. Our hypothesis is that the constraints on priming highlighted through such structures should also hold for other rare constructions (with no crossing dependencies). This is because, while there is some evidence for crossing dependencies to be difficult to process due to its inherent complexity (Husain and Vasishth, 2015), there is also evidence that frequency can explain the comprehension difficulty of crossing dependencies (Levy et al., 2012). The fact that crossing dependencies need not be inherently difficult to process was demonstrated by Bach et al. (1986) who showed that native speakers of Dutch found cross-serial dependencies in Dutch (that are crossing) more acceptable compared to German speakers who read matched set of embedded constructions in German (that are not crossing). So, theoretically, it is quite reasonable to assume that the processing effects in sentences with crossing dependencies can be explained through a frequency-based approach. Future work will be required to tease apart the influence of frequency vs inherent structural complexity on syntactic priming.

### 6.2. Priming or Prediction

It has been suggested that syntactic priming is more of a passive process that relies on lingering activations of the recently attended linguistic representations, while syntactic prediction is more of an active process where bottom-up input is used to actively preactivate unseen linguistic representations in a top-down fashion (Kuperberg and Jaeger, 2016). Similar arguments have been propounded by Huettig (2015) (also see, Kuperberg, 2007) who equates priming and prediction to be two distinct systems (following, Kahneman, 2011). The results of our experiment could be interpreted to support such a proposal. It could be the case that these two processes are distinct—while syntactic priming can be understood in terms of inter-sentential structural activation, syntactic prediction could be driven by intra-sentential syntactic cues.

However, one way to reconcile our results with a residual activation-based proposal (Pickering and Branigan, 1998) would be to assume that activation of linguistic structures is influenced not only by inter-sentential linguistic factors but also intra-sentential linguistic cues. Something similar was proposed (but not tested) in Reitter (2008) where spreading activation was used to formalize short-term priming using the ACT-R framework (Anderson et al., 2004).

### 6.3. Factors Influencing Priming

Syntactic priming during comprehension has been argued to require lexical overlap between the prime and the target. Results from Experiment 3 show that this is not a sufficient precondition; the SPR experiment showed that inspite of the lexical overlap, Hindi native speakers faced processing difficulty in certain conditions. In addition, the pilot sentence completion study (Experiment 2a) provides preliminary evidence that non-lexical factors might also play a role during priming. In particular, our results show that there was an increase in the use of transitive non-finite verb in the presence of an intransitive non-finite verb. For example, use of the intransitive non-finite verb *hasnaa* ‘laughing' led to the increase of a transitive non-finite verb *maarnaa* ‘hitting' due to the presence of an ACC case-marker. While the two verbs do not share the sub-categorization information, they do share certain morphological information; the *=naa* ending on both these verbs signifies a infinitival phrase (Kachru, 2006). The result of the pilot study needs to be replicated with a larger study. If correct, this would suggest that certain morphological (non-lexical) information could also be primed in certain context. This result is compatible with recent finding on graded predictability (Staub, 2015; Luke and Christianson, 2016). We intend to investigate this in the future.

Priming is also known to be influenced by frequency of the prime structure in both production (e.g., Hartsuiker and Kolk, 1998; Scheepers, 2003) as well as comprehension (e.g., Wei et al., 2016). In particular, it has been found that less frequent primes have stronger priming effect compared to more frequent primes (known as the *inverse frequency effect*). Relatedly, syntactic priming has also been looked at from the perspective of learnability and prediction error (e.g., Jaeger and Snider, 2013; Myslín and Levy, 2016). This line of work has investigated the role of complexity of the prime structure and its effect on target production and comprehension. In our work we have kept the structural complexity of the prime constant while manipulating the complexity of the target structure (cf. Table 3). As far as learnability of such complex target structures is concerned, we found no such effect in the *post-hoc* analysis discussed in Experiment 3. At the same time, we did find a global facilitatory effect of item exposure.

Our work highlights the role of *structural complexity of the target* with regard to syntactic priming during comprehension. This constraint on priming goes beyond previously investigated constraints such as lexical overlap, complexity/frequency of the prime, etc. Given the proposals that equate processing constraint during comprehension to production processes (e.g., MacDonald, 2013), exploring the role of this constraint on syntactic priming during production will be taken up in the future.

## 7. Conclusion

Results from a series of sentence completion studies show that syntactic priming is affected by the probability of using the prime structure in the target sentence. When the linguistic cues (e.g., case-markers) from the target and the prime structure form a rare structure, the strength of the priming effect gets attenuated; otherwise, the prime structure persists in the target. In our studies, integrating an Accusative/Ergative case-marked noun in the target sentence with the intransitive non-finite prime structure lead to an infrequent structure, while integrating a Locative case-marked noun in the target sentence with the prime lead to a relatively frequent structure. The completion results were found to be consistent with the reading time results obtained in an SPR study where RTs were longer in the target sentences with Accusative/Ergative case-markers compared to the condition with the Locative case.

Our work highlights a novel constraint on syntactic priming in an SOV language like Hindi. In particular, it demonstrates the role of preverbal linguistic cues (here, case-markers) during syntactic priming. The results suggest that the prime structure is continuously evaluated against the target linguistic cues. If this integration leads to a rare structure then the chances of persisting with the prime structure in the target reduces.

## Data Availability Statement

The data and the analysis files for this study can be found at https://doi.org/10.17605/OSF.IO/M9BPN.

## Ethics Statement

The studies involving human participants were reviewed and approved by IIT Delhi Institute Ethics Committee, IIT Delhi, India (web.iitd.ac.in/~elangovan/ethics/ethics.htm). The patients/participants provided their written informed consent to participate in this study.

## Author Contributions

SH and HY conceived and designed the experiments, analyzed the data, analysis tools, and wrote the paper. HY contributed materials and performed the experiments.

### Conflict of Interest

The authors declare that the research was conducted in the absence of any commercial or financial relationships that could be construed as a potential conflict of interest.

## Footnotes

^1^https://www.ethnologue.com/statistics/size.

^2^We follow the established norms in the literature in deciding the number of participants. For the completion study, ~30 is the norm, for example, see Experiments 1, 3, and 4 in Pickering and Branigan (1998). For self-paced reading experiments, ~60 is the norm, for example, see Vasishth and Lewis (2006). For pilot/norming study 10–15 is the norm, for example, see Husain et al. (2014). On similar lines, we have used 28 and 27 participants in the completion studies discussed in Experiment 1 and 2 respectively. The SPR study discussed in Experiment 3 has 75 participants.

^7^In Hindi, an intransitive verb cannot take an Accusative/Ergative marked noun as its argument.

^9^Prime-Target Structural Compatibility Hypothesis: ‘During comprehension, syntactic priming is stronger when the linguistic cues of the target and the prime structure combine to form a frequent structure, otherwise priming effect will be weak.'

^10^Recall that the ACC case can be incorporated in the NFV clause, but by positing a *transitive/causative* non-finite verb.

^11^Recall that, in a Hindi dependency treebank (Bhatt et al., 2009), only 1% of the total 6,342 non-finite clause instances were found to have a crossing dependency.

^13^As noted earlier, such a usage forms a non-crossing dependency with the ACC case-marked noun acting as the object of the *transitive* non-finite verb.

^15^Thanks to a reviewer for this alternative explanation.
